# Spatial and temporal analysis of hospitalized dengue patients in Bandung: demographics and risk

**DOI:** 10.1186/s41182-021-00329-9

**Published:** 2021-05-26

**Authors:** Lia Faridah, I. Gede Nyoman Mindra, Ramadhani Eka Putra, Nisa Fauziah, Dwi Agustian, Yessika Adelwin Natalia, Kozo Watanabe

**Affiliations:** 1 Parasitology Division, Department of Biomedical Science, Faculty of Medicine, Universitas Padjadjaran, Bandung, Indonesia; 2 Foreign Visiting Researcher at Department of Civil and Environmental Engineering, Ehime University, Matsuyama, Japan; 3 Department of Statistics, Universitas Padjadjaran, Bandung, Indonesia; 4 School of Life Science and Technology, Institut Teknologi Bandung, Jl. Ganeca 10, Bandung, West Java 40132 Indonesia; 5 Department of Public Health, Faculty of Medicine, Universitas Padjadjaran, Bandung, Indonesia; 6 Department of Civil and Environmental Engineering, Ehime University, Matsuyama, Japan

**Keywords:** Bandung, Dengue infection, Spatial pattern

## Abstract

**Background:**

Bandung, the fourth largest city in Indonesia and capital of West Java province, has been considered a major endemic area of dengue, and studies show that the incidence in this city could increase and spread rapidly. At the same time, estimation of incidence could be inaccurate due to a lack of reliable surveillance systems. To provide strategic information for the dengue control program in the face of limited capacity, this study used spatial pattern analysis of a possible outbreak of dengue cases, through the Geographic Information System (GIS). To further enhance the information needed for effective policymaking, we also analyzed the demographic pattern of dengue cases.

**Methods:**

Monthly reports of dengue cases from January 2014 to December 2016 from 16 hospitals in Bandung were collected as the database, which consisted of address, sex, age, and code to anonymize the patients. The address was then transformed into geocoding and used to estimate the relative risk of a particular area’s developing a cluster of dengue cases. We used the kernel density estimation method to analyze the dynamics of change of dengue cases.

**Results:**

The model showed that the spatial cluster of the relative risk of dengue incidence was relatively unchanged for 3 years. Dengue high-risk areas predominated in the southern and southeastern parts of Bandung, while low-risk areas were found mostly in its western and northeastern regions. The kernel density estimation showed strong cluster groups of dengue cases in the city.

**Conclusions:**

This study demonstrated a strong pattern of reported cases related to specific demographic groups (males and children). Furthermore, spatial analysis using GIS also visualized the dynamic development of the aggregation of disease incidence (hotspots) for dengue cases in Bandung. These data may provide strategic information for the planning and design of dengue control programs.

## Introduction

Dengue infection has been considered a significant global health problem in many tropical and subtropical countries, with 3.97 billion people worldwide are at risk [1, 2]. Indonesia is among many Southeast Asian countries that are considered hyperendemic [3], with an estimation of 600,000 cases of dengue annually, of which approximately 180,000 lead to hospitalization [4]. One of the regions with the highest incidence of dengue cases per year is Bandung City where the total number has steadily increased from 4000 in 2008 to 6000 in 2013 [5, 6].

However, these incidence figures may actually be an underestimate due to dependence on passive surveillance systems. Passive surveillance systems depend largely on a country’s capacity or resources (e.g., clinical skill and laboratory resources) to identify all cases of a disease. When a country does not possess the required capacity or resources, passive surveillance is less likely to detect all cases, which could lead to periodic outbreaks [7–9]. Considering the lack of a reliable dengue surveillance system in Indonesia [10], we propose the use of spatial pattern analysis of a possible outbreak of dengue cases, such as through the Geographic Information System (GIS), based on confirmed dengue cases reported by medical institutions. This approach could help communities and policymakers by providing strategic information without the requirement for extensive capacity or resources.

In recent years, GIS has been utilized to assess, identify, and visualize the potential risk factors involved in disease transmission [9, 11–14]. Employed by many agencies of public health epidemiology, this technology is considered to be a powerful tool in addressing epidemiological problems and providing spatial models for improving the effectiveness of interventions for various infectious diseases, such as black fever, diarrhea, typhoid, malaria, and dengue [15–18]. In these studies, spatial analysis and statistics such as spatial autocorrelation have been used to develop models of spatial distribution and patterns of disease that demonstrate the spatial clusters of the disease incidence (”hotspots”) [13]. Hotspots can be identified as a spatial cluster of high value, in this case, an area with a record of high dengue incidences that can be assumed to be where dengue outbreaks are most likely to occur [19–22]. The application of cluster analysis has provided a better understanding for some cases of dengue, such as (1) the dispersion of the first epidemic of dengue virus serotype in Vitoria, Brazil, as cluster analysis showed the speed of dispersion was highest in the hotspots [23]; (2) the dynamics of dengue cases of Andhra Pradesh, India, as the cluster analysis classified 23 districts into 3 major clusters based on the risk of dengue incidences and 4 districts as the major hotspots [20]; and (3) the pattern of dengue exposure based on the types of the dengue virus in Singapore [24].

Taking into account the lack of reliable surveillance networks in a developing country, spatial epidemiological investigations can be approached by mapping the relative risk, a process which essentially compares the probability of disease in the exposed group to the probability in the unexposed group [25], using the patient data of reported positive cases. A previous study employing this approach in Bucaramanga, a smaller Indonesian city with a low population density, was reasonably successful in estimating and mapping the relative risk of dengue incidence [26]. However, our study might produce different dynamics, as Bandung has a much bigger area (Bandung covers an area of more than 167.29 km^2^ while Bucaramanga covers only 27 km^2^). This larger space may lead to broader movement of disease carriers among areas in Bandung.

Furthermore, dengue incidences have a temporal signature, as outbreaks at a particular space-time location can surge and resurface in a cyclic pattern. It is therefore critical to analyze the data through space-time clustering—as the disease tends to exhibit in a non-random pattern—and geosurveillance—as the disease tends to occur in similar regions which could translate into geographical position [27, 28]. However, there are two important concerns. First, uncertainty exists regarding geographic context [29], caused by spatial uncertainty of the areas which, in the case of geospatial error, is influenced by the people being studied. Case reports are based mostly on residential or work address, both of which have a dynamic nature. Second, a possibility of conclusion biases [28] is linked to the temporal dimension, as variability may occur in the time between the occurrence of early symptoms and the report of a positive dengue case) [30]. Resulting conclusion biases may lead to errors in the estimation of the risk of dengue incidences in the form of either underestimation or overestimation of the local risk of the diseases [31–33], with the consequences of misevaluation of the spatial data and policymakers’ receiving biased evidence.

The high uncertainty which leads to conclusion biases can be overcome by applying higher and optimal kernel bandwidth [34] which is estimated by kernel density estimation [30]. Kernel density estimation is a non-parametric way to estimate the probability density function of a random variable based on a finite data sample. A curve of distribution is created by weighing the distance of all points in each specific location along with the distribution. As part of GIS analysis, kernel density estimation generates a summary of the spatial distribution (based on the point event when the disease occurred or is reported) in which each grid cell in the geographical positional map reflects the intensity of the corresponding process (e.g., disease incidence, medical cases, accident) at that location [35–37]. This method can identify the change of localized risk which is usually blurred in the aggregated data of a regional count [38, 39]. Further, it delimits the spatial extent of disease occurrences, allowing less computational time and thus reducing the time required to develop a dengue prevention strategy [30, 40].

Through this study, we aimed to develop spatial-temporal patterns of dengue case incidence by the application of the kernel density estimation to assess the spatial distribution of relative dengue risk in Bandung over a specific range of years. The study also enabled us to produce a map visualizing the relative risk of dengue in Bandung by indicating the hotspots of dengue transmission. This research improves on previous studies about the pattern of dengue spread which simply showed the total dengue incidence for a specific time without comparison to the number of cases in other areas, or without showing spatial and temporal fluctuations [6]. Furthermore, information on the location of hotspots may help policymakers to direct vector eradication activities as well as to plan more suitable strategies for the prevention of outbreaks and early warning information, including for communities to encourage individuals in reducing the risk of dengue cases in their region.

## Methodology

### Study area

Bandung is the largest city on the southern island of Java, located at 107^o^36′ east longitude and 6^o^55′ south latitude. It has a total area of 16,729.65 hectares and lies at 791 m above sea level (asl). Its highest point is in the northern area at 1050 m asl, while the lowest point is located in the southern area at 675 m asl. The southern part of the city consists mostly of sloping contours, while the northern part is far more mountainous. The city comprises 30 sub-districts with 151 villages and can be divided as north, south, east, and west boundaries [41].

### Dengue epidemiological data

Monthly reports of diagnosed dengue cases from 16 hospitals in Bandung were collected from the Dengue Disease Surveillance database of the city health office. These reports were obtained for the period January 2014 to December 2016. Dengue cases were distinguished into five different categories based on symptoms and laboratory results (number of hemoglobin, leukocyte, thrombocyte, hematocrit, and NS1), namely undifferentiated fever, dengue fever, dengue hemorrhagic fever, dengue shock syndrome, and expanded dengue syndrome [42]. Undifferentiated fever is a simple fever similar to other viral infections that may be observed among patients who have been infected with dengue before, in particular for the first time (i.e., primary infection). Expanded dengue syndrome is a category of unusual manifestations with the involvement of dengue-associated symptoms in several organs such as the liver, kidneys, brain, or heart. In this study, we used confirmed dengue hemorrhagic fever cases only.

The demographic profile of each reported case included the patient’s age, gender, residential address, date of confirmed diagnosis, and reporting hospital. The data were analyzed anonymously and it was assumed that each infection occurred at the residential address of the patients. Population size data of 151 villages of Bandung for 2014–2016 were also obtained from the Bandung Central Bureau of Statistics for the year 2017. Data were further categorized by age group (Table 1). All of these data were then statistically analyzed using ANOVA and t-tests. All statistical testing was carried out by R-Studio.

**Table 1:** Age groups of dengue patients

Age	Category
Less than 1 year old	Infancy
1–14 years old	Youth
15–24 years old	Young adulthood
25–44 years old	Middle adulthood
45–64 years old	Older adulthood
More than 65 years old	Retirement

### Kernel density estimation for relative risk

Using the dengue epidemiological data from 2014 to 2016, we employed the kernel smoothing method [43, 44] to estimate a continuous spatial distribution of the relative risk of dengue incidences which we defined as the probability of the cases occurring during the period of dengue outbreak.

The probability was estimated from the spatial distributions of dengue cases (the residence address of the patients with confirmed dengue) and the temporal distribution of dengue cases at each area within Bandung (the time when the positive cases were reported). By defining the spatial distribution of dengue cases (*f*) as the spatial density function, and distribution of infected population at specific regions (*g*) as the temporal density function, then the dengue relative risk at spatial coordinate $$ \overrightarrow{z}=\left(\begin{array}{c}x\\ {}y\end{array}\right)\epsilon W $$ (*W* is Bandung) can be defined as

$$ r\left(\overrightarrow{z}\right)=\frac{f\left(\overrightarrow{z}\right)}{g\left(\overrightarrow{z}\right)} $$

Suppose $\overrightarrow{d_i}$ and $\overrightarrow{p_j}$ is a spatial coordinate for the *i-*th dengue case and a spatial coordinate for the *j-*th individual. Suppose *m* and *n i* are the number of dengue cases and the number of the total population in a year, respectively. Therefore, density function *f* and *g* can be defined as

$$ f\left(\overrightarrow{z}\right)\approx \hat{f}\left(\overrightarrow{z}\right)=\frac{1}{m}\sum \limits_{i=1}^m{K}_h\left(\overrightarrow{z}-{\overrightarrow{d}}_i\right) $$

$$ g\left(\overrightarrow{z}\right)\approx \hat{g}\left(\overrightarrow{z}\right)=\frac{1}{n}\sum \limits_{j=1}^n{K}_h\left(\overrightarrow{z}-{\overrightarrow{p}}_j\right) $$

A variable bandwidth selector was used in the multivariate kernel density estimation [45], which is represented in the following equation:

$$ {K}_h\left(\overrightarrow{w}\right)=\frac{1}{h_x{h}_y}K\left(\frac{\overrightarrow{w}}{h}\right)=\frac{1}{h_x{h}_y}\frac{1}{2\pi}\mathit{\exp}\left(\frac{-1}{2}{\overrightarrow{w}}^T{\left({\left(\begin{array}{cc}{h}_x& 0\\ {}0& {h}_y\end{array}\right)}^{-1}\right)}^2\overrightarrow{w}\right) $$

where $$ \left(\begin{array}{cc}{h}_x& 0\\ {}0& {h}_y\end{array}\right) $$ is the scaled bandwidth.

The optimum bandwidth used in this study was normal smoothing, which is the minimum asymptotic mean integrated squared error [37], calculated using the normal scale (NS) function in the sparr package of Rstudio. We employed normal smoothing to provide asymptotically optimal fixed bandwidths for spatial or spatiotemporal normal densities that showed for dengue incidences for each observation year.

## Results

### Hospitalized dengue cases

**Fig. 1:**
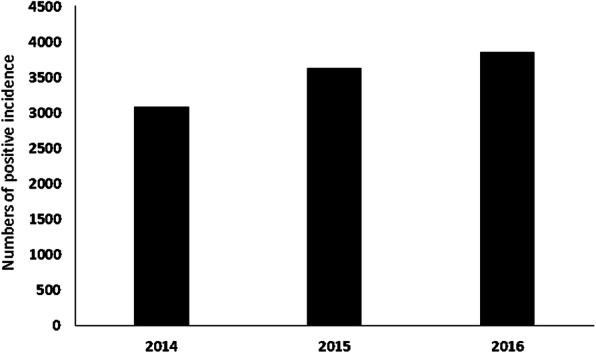
Number of dengue cases reported from the 16 local hospitals in Bandung from 2014 to 2016. Hotspot analysis helps in identifying clustered data points on a map. Therefore, using this method permits the averaging of the results, based on cumulative monthly averaging or seasonal averaging

**Fig. 2:**
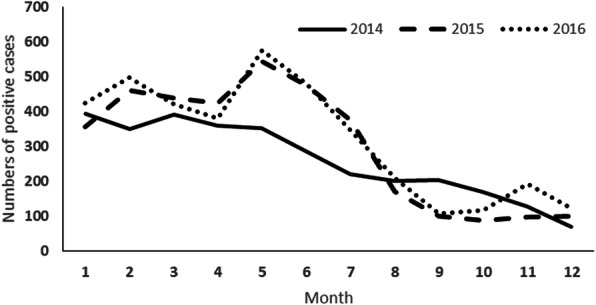
Monthly dengue cases were reported from the local hospital in Bandung from 2014 to 2016

A total of 10,573 hospitalized cases were registered from January 2014 to December 2016, with an annual increase rate of 11.83% (Fig. 1). In 2014, the peak of dengue incidence happened in January and March, while for 2015 and 2016 the peak happened during the dry season (Fig. 2)*.*

**Fig. 3:**
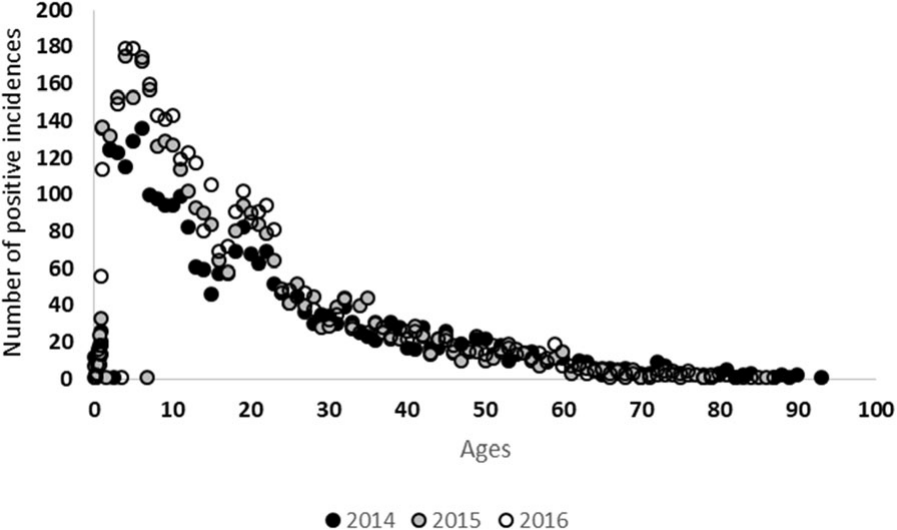
Age distribution of patients with dengue incidence reported by Bandung hospitals 2014–2016

**Fig. 4:**
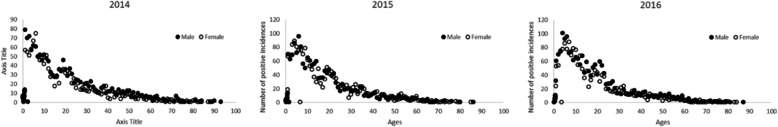
Gender breakdown of age distribution of patients with dengue incidence reported Bandung hospitals 2014–2016

**Fig. 5:**
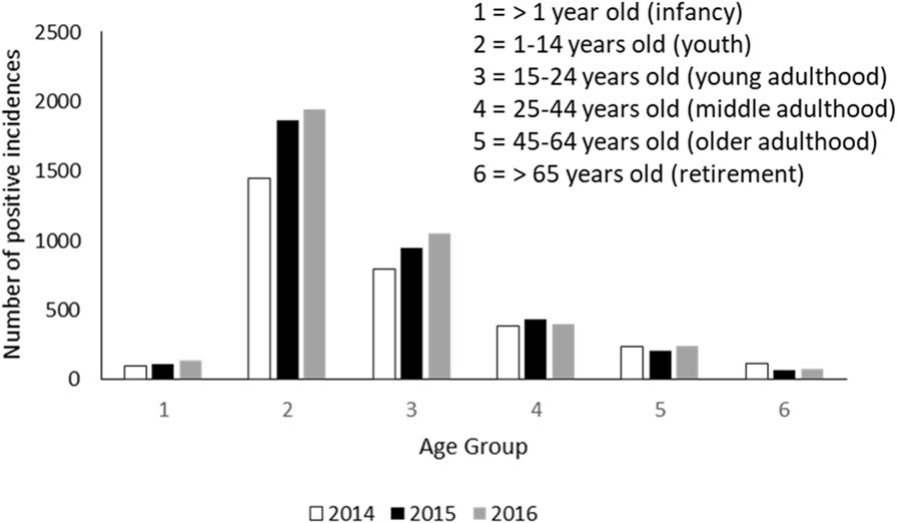
Distribution of cases among age groups reported by Bandung hospitals 2014–2016

In general, most patients (on average 42.15 ± 1.4% for 3 years data) with reported dengue cases were children under 10 years old for all observation years, with the total number of patients consistently declining with age (Fig. 3). The pattern was relatively constant among the three observation years (ANOVA, *p = 0.283*). Also, more male (*n* = 5533) than female (*n* = 5040) dengue patients were reported during the observation period (Fig. 4), although the difference was not statistically significant (*t* test, *p = 0.31*). Annually, there were increasing trends of positive cases for young patients (Fig. 5) alongside a decreasing pattern of cases for an older, including much older, population (15 to 65 years old).

### Dengue risk estimation and spatial representation

Following normal smoothing calculation, optimal bandwidth for each observational year was estimated as follows (Table 2):

**Table 2:** Bandwidth value under normal smoothing

	Normal smoothing
Dengue cases 2014	*h* = 915.34 m
Dengue cases 2015	*h* = 906.49 m
Dengue cases 2016	*h* = 907.20 m

**Fig. 6:**
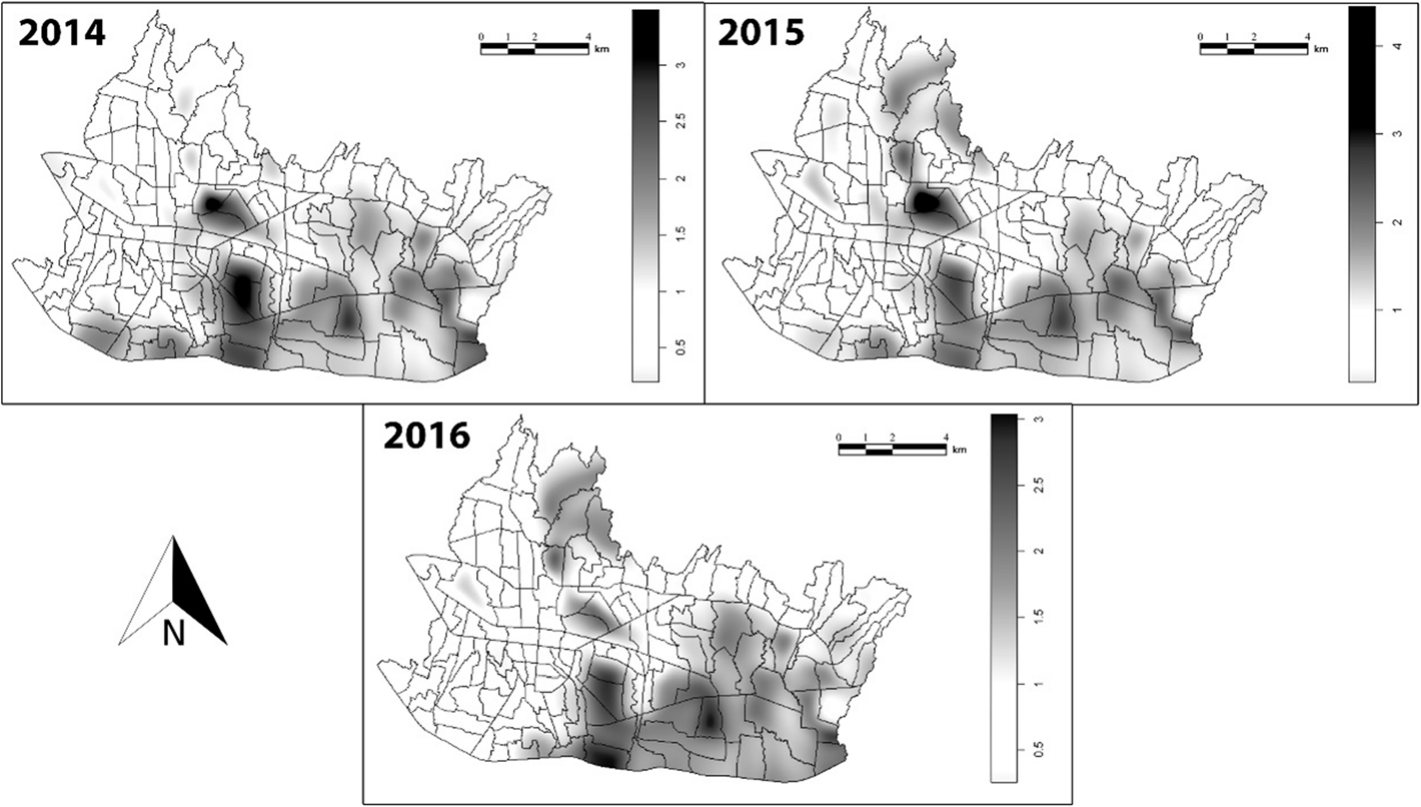
Spatial analysis of the relative risk of dengue in Bandung at the village level in **a** 2014, **b** 2015, and **c** 2016

Further analysis employed the bandwidth calculation to estimate the relative risk and subsequently to map the risk into a spatial representation. The pattern of the relative risk of dengue in Bandung did not change significantly between 2014 and 2016. In general, villages with high relative dengue risk were more concentrated in the southern and southeastern regions of the city, while villages with low relative dengue risk were mostly found in the western and northeastern regions (Fig. 6). However, it is observable that a new aggregation of the disease incidence (hotspots) for dengue developed in the northern area of Bandung in 2015.

Despite the change in 2015, the hotspots were relatively constant over time, with the majority found in southeastern areas over time. To estimate the relative risks over space and time we take into account the population at risk. The basic formulation of the relative risk is defined as:

$$ {\boldsymbol{\theta}}_{\boldsymbol{it}}=\frac{{\boldsymbol{y}}_{\boldsymbol{it}}}{{\boldsymbol{E}}_{\boldsymbol{it}}};\boldsymbol{i}=\mathbf{1},\dots, \mathbf{30}\ \mathbf{and}\ \boldsymbol{t}=\mathbf{2014},\mathbf{2015},\mathbf{2016} $$

where ***θ***_***it***_, ***y***_***it***_ and ***E***_***it***_ denote the relative risk, number of incidences, and expected rate at area *i* and time *t,* respectively. The expected rate ***E***_***it***_ is defined as [46]

$$ {\boldsymbol{E}}_{\boldsymbol{i}\boldsymbol{t}}={\boldsymbol{N}}_{\boldsymbol{i}\boldsymbol{t}}\frac{\sum_{\boldsymbol{i}=\mathbf{1}}^{\boldsymbol{n}}{\sum}_{\boldsymbol{t}=\mathbf{1}}^{\boldsymbol{T}}{\boldsymbol{y}}_{\boldsymbol{i}\boldsymbol{t}}/\boldsymbol{nT}}{\sum_{\boldsymbol{i}=\mathbf{1}}^{\boldsymbol{n}}{\sum}_{\boldsymbol{t}=\mathbf{1}}^{\boldsymbol{T}}{\boldsymbol{N}}_{\boldsymbol{i}\boldsymbol{t}}/\boldsymbol{nT}} $$

with ***N***_***it***_ as the population at risk at area *i* and time *t*.

According to this formulation, the relative risks were estimated by taking into account the population at risk.

In this paper, we used the normal kernel density approach. “The optimum bandwidth used in this study was normal smoothing, which is the minimum asymptotic mean integrated squared error [37], calculated using the normal scale (NS) function in the sparr package of Rstudio to provide the asymptotically optimal fixed bandwidths for spatial or spatiotemporal normal densities that were shown for dengue incidences for each observation year.”

The local Moran’s *I* statistics is commonly used to identify hotspots. It is able to estimate density distribution of events at the local level, and identify statistically significant hot spots in a dataset. However, very often areas identified as hotspots from LISA results are not grouped into a high-density threshold on a kernel density estimation map. When analyzing and mapping spatial patterns (e.g., dengue incidence), it is important to ensure that the identification of hotspots is as accurate and effective as possible [47].

Underreported data in disease study is difficult to avoid. However, by assuming the underreporting occurs in all areas and times randomly, we believe that the underestimation problem is not an issue due to random error assumption.

## Discussion

### Dengue cases and seasonal changes

Dengue incidences in Bandung showed an increasing number from 2014 to 2016. In general, the pattern of hospitalized cases was remained relatively stable despite the change in hotspot areas. The pattern showed large numbers of cases early in the year followed by a peak period about May to June. The pattern is strongly related to meteorological factors such as rainfall and humidity which can been observed at different scope levels: village-wide (Faridah, unpublished data), region-wide (West Java province and its nearby areas) [48], and nationwide (other regions in Indonesia) [49–51] and consistent with reports from other regions of the world even though an effect of temperature is relatively insignificant in our study area as in other tropical countries [52–54].

### Demographic analysis of hospitalized dengue cases in Bandung, West Java

This study also found that most of the patients were male, similar to previous studies from Cambodia, Malaysia, Sri Lanka, Singapore, the Philippines, and India [55–57]. One contribution to this finding might be the higher number of male workers in government offices and private enterprises in Bandung [58]. Thus, there could be a higher likelihood for males to be exposed to dengue-carrying mosquitoes during working hours (mostly daytime) at the workplace or while traveling to and from their workplace [57]. Another contributing factor may be a gender difference in susceptibility to certain serotypes, For example, Chang et al. (2018) [59] reported that males are more likely to be infected with DENV-1, and that dengue cases in females are mostly caused by DENV-3. In Bandung, DENV-1 is more commonly found than DENV-3 [60, 61]. Although to our knowledge, our study is the first to include information on the gender pattern of dengue cases, and our data did not include information on patient serotype. Thus, further studies are required to examine the higher incidence rate in males and particularly the relationship to serotype

The study also highlighted a strong demographic pattern of cases in children, with a significant increase in 2016. This result is similar to other data from a different city in West Java [48] which suggests that the pattern might occur across West Java. Furthermore, children under 10 years old were at the highest risk of dengue infection, which supports findings in the Philippines and Brazil that suggested a possible relation between age and an increase in immunity [62].

### Spatial analysis of hospitalized dengue cases in Bandung, West Java

Our results confirmed that dengue cases in Bandung have a clustered distribution pattern among the local villages. High-risk areas of dengue incidences were mostly located in Bandung’s downtown, in the intersection of the area between Cibeunying, Karees, Tegalega, and Bojanegara. This area is one of the most economically productive in Bandung, where most of the best public services (education, government, health), industries, commercial enterprises, and recreation are concentrated and the rate of population growth is considered higher than in other regions due to a higher birth rate and urbanization [63]. This result supports previous studies that showed dengue incidence correlated with urban growth and urbanization [64–69].

The kernel density estimation allowed the generated map to show the development of new hotspots in the northern part of the city since 2015. This was possible because the estimation process adds the pattern of point event changes in an equation and compares it to other point events. Thus, although the number of cases may be much lower than in other regions, a positive change will be registered as a positive result of forecasting—in this case, showing an increase in the risk of dengue incidences. A possible explanation of the phenomenon may be related to the rapid development of the northern part of the city during the last 10 years. The development activities created a region that provides better education, job opportunities, recreation, and standard of living that have encouraged temporary rural-urban immigration from established hot spots [70]. This result is supported by similar studies in Thailand [71] and Brazil [72] that showed a significant impact of human movement on the transmission of dengue in a local area. While this study did not calculate the exact distance of each clustered dengue risk coordinate, the clusters shown in Fig. 6 did not span widely beyond the epicenters of the risk. This suggests that dengue transmission dynamics are limited to a small scale. Guzetta et al. (2018) [73] found that a large proportion of cases were transmitted via short-distance human movement (< 1 km) with a limited contribution of long-distance commuting within the city. Moreover, research conducted in Venezuela showed dengue infection often clustered in a smaller range which was within and around households and blocks in radius 20–110 m [14]. Dengue virus transmission is spatially heterogeneous because the dynamics are determined by a complex interplay between environmental and climatic factors, abundance and competence of vector species, human density and behavior, ecological interactions among viral strains, and profiles of immunity in the population [74, 75].

Another notable point in the generated spatial map (Fig. 6) is the annual changes of cluster size, even though the precise fluctuations of cluster size were not measured in this study. Further study is needed to confirm and analysis this phenomenon as it might also reflect inconsistency in dengue programs or underreporting of dengue cases. Previous studies found inherent inadequacy and inaccuracy in Bandung hospitals’ systems for reporting to the Bandung City Health Office. These studies suggested that the reporting system for dengue in Bandung should be evaluated and strengthened to minimize the number of unreported cases [6, 76]. Inaccuracy might also be one reason why no clusters of dengue risk were detected in the western part of Bandung.

The main limitation of this study is a broader inability to assess the underreporting of hospitalized patients’ data. The data we gathered from Bandung City Health Office covered only 60% of the hospitals that were registered in Bandung. The extent to which such partial data might have influenced the analysis result is unknown. However, the omissions are considered minor and the findings still applicable, as we gathered most of the data from type B hospitals, which generally act as the primary reference for health services in their respective areas.

A study by Tapia-Coyer is relevant to our findings. It showed that community empowerment is a key aspect of disease-eradicating strategy, as it allows a local population to drive the eradication of diseases in its environment. Hence, a sustainable process is necessary to encourage individuals to maintain efforts in keeping their environment free of dengue [74]. Knowledge of an area’s dengue risk may further enhance community members’ efforts to prevent dengue preventing contact with the vector and ultimately eradicating it.

## Conclusion

This study demonstrates a strong pattern of reported cases related to specific demographic groups (males and children). Furthermore, spatial analysis using GIS also visualized the dynamic development of aggregation of the disease incidence (hotspots) for dengue cases in Bandung. In the future, observation of clustered hotspots in Bandung could provide strategic information for the planning and design of a dengue control program and may encourage communities to initiate their own dengue prevention programs. Existing surveillance methods should be evaluated and modified as needed based on the spatial analysis presented in this report so that local resources can be optimally utilized in a specific area. Moreover, the clustering distance information, which is around 900 m in radius, and demographic data can be used for detailed technical planning in dengue case countermeasures.

## Data Availability

The data that support the findings of this study are available from Dinas Kesehatan Kota Bandung (the Health Service of Bandung City) but restrictions apply to the availability of these data, which were used under license for the current study and therefore are not publicly available. Data are, however, available from the authors upon reasonable request and with permission of Dinas Kesehatan Kota Bandung (The Health Service of Bandung City).

## Abbreviations

ANOVA: Analysis of variance
asl: Above sea level
DENV: Dengue virus
DF: Dengue fever
DHF: Dengue hemorrhagic fever
GIS: Geographic Information System
RNA: Ribonucleic acids

## References

[1] Ayukekbong JA, Oyero OG, Nnukwu SE, Mesumbe HN, Fobisong CN (2017). Value of routine dengue diagnosis in endemic countries. World J Virol.

[2] World Health Organization (2012). Global strategy for dengue prevention and control 2012–2020.

[3] Maula AW, Fuad A, Utarini A (2018). Ten-years trend of dengue research in Indonesia and South-east Asian countries: a bibliometric analysis. Global Health Action.

[4] Hasan S, Jamdar SF, Alalowi M, Al Beaiji SM (2016). Dengue virus: A global human threat: Review of literature. J Int Soc Prev Community Dent.

[5] Bandung City Health Office (2014). Bandung Health Profile 2013.

[6] Titik R, Ardini R, Heni D, Asep S. Spatial distribution of Dengue Haemorhagic Fever in urban setting of Bandung City. Global Med Health Commun. 2017. 10.29313/gmhc.v5i3.2535.

[7] Chao DY, Lin TH, Hwang KP, Huang JH, Liu CC, King CC (2004). 1998 dengue hemorrhagic fever epidemic in Taiwan. Emerging Infect Dis.

[8] Fuentes-Vallejo M (2017). Space and space-time distribution of dengue in a hyper-endemic urban space: the case of Girardot, Colombia. BMC Infect Dis.

[9] Kao JH, Chen CD, Tiger Li ZR, Chan TC, Tung TH, Chu YH, Cheng HY, Liu JW, Shih FY, Shu PY, Lin CC, Tsai WH, Ku CC, Ho CK, King CC (2016). The critical role of early dengue surveillance and limitations of clinical reporting – implications for non-endemic countries. PLOS ONE.

[10] Faridah L, Rinawan F, Fauziah N, Mayasari W, Dwiartama A, Watanabe K (2020). Evaluation of Health Information System (his) in the surveillance of Dengue in Indonesia: Lessons from case in Bandung, West Java. Int J Environ Res Public Health.

[11] Arsalan Q, Qadar LT, Ochani RK, Tahir F, Majid Z (2019). Collateral presentation of Malaria and Dengue viral Hemorrhagic fever: a rare case. Cureus.

[12] Kakarla SG, Caminade C, Mutheneni SR, Morse AP, Upadhyayula SM, Kadiri MR, Kumaraswamy S (2019). Lag effect of climatic variables on dengue burden in India. Epidemiol Infect.

[13] Pan CY, Liu WL, Su MP, Chang TP, Ho HP, Shu PY, Huang JJ, Lin LJ, Chen CH (2020). Epidemiological analysis of the Kaohsiung city strategy for dengue fever quarantine and epidemic prevention. BMC Infect Dis.

[14] Vicente CR, Herbinger KH, Junior CC, Romano CM, Cabidelle ASA, Froschl G (2017). Determination of clusters and factors associated with dengue dispersion during the first epidemic related to Dengue virus serotype 4 in Vitória, Brazil. PLoS ONE.

[15] Phung D, Huang C, Rutherford S, Chu C, Wang X, Nguyen W, Nguyen NH, Do CM, Nguyen TH (2015). Temporal and spatial patterns of diarrhoea in the Mekong Delta area, Vietnam. Epidemiol Infect.

[16] Dewan A, Abdullah AY, Shogib MR, Karim R, Rahman MM (2017). Exploring spatial and temporal patterns of visceral leishmaniasis in endemic areas of Bangladesh. Trop Med Health.

[17] Corner RJ, Dewan AM, Hashizume M (2013). Modelling typhoid risk in Dhaka Metropolitan Area of Bangladesh: the role of socio-economic and environmental factors. Int J Health Geograph.

[18] Wangdi K, Canavati SE, Ngo TD, Nguyen TM, Tran LK, Kelly GC, Martin NJ, Clements ACA (2020). Spatial and temporal patterns of malaria in Phu Yen Province, Vietnam, from 2005 to 2016. Am J Trop Med Hygiene.

[19] Majid NA, Nazi NM, Mohamed AF (2019). Distribution and spatial pattern analysis on dengue cases in Seremban District, Negeri Sembilan, Malaysia. Sustainability.

[20] Mutheneni SR, Mopuri R, Naish S, Gunti D (2018). Upadhyayul. Spatial distribution and cluster analysis of dengue using self organizing maps in Andhra Pradesh, India, 2011–2013. Parasit Epidemiol Contr.

[21] Pessanha JEM, Caiaffa WT, Almeida MCM, Brandao ST, Proietti FA (2012). Diffusion pattern and hotspot detection of dengue in Belo Horizonte, Minas Gerais, Brazil. J Trop Med.

[22] Wangdi K, Clements ACA, Du T, Nery SV (2018). Spatial and temporal patterns of dengue infections in Timor-Leste, 2005–2013. Parasites Vectors.

[23] Vicenti-Gonzalez MF, Grillet ME, Velasco-Salas ZI, Lizarazo EF, Amarista MA, Sierra GM, Comach G, Tami A (2017). Spatial Analysis of Dengue Seroprevalence and Modeling of Transmission Risk Factors in a Dengue Hyperendemic City of Venezuela. PLoS Neglect Trop Dis.

[24] Sangkaew S, Tan LK, Ng LC, Ferguson NM, Dorigatti I (2020). Using cluster analysis to reconstruct dengue exposure patterns from cross-sectional serological studies in Singapore. Parasites Vectors.

[25] Tenny S, Hoffman MR (2020). Prevalence. StatPearls.

[26] Martínez-Bello DA, López-Quílez A, Torres PA (2017). Relative risk estimation of dengue disease at small spatial scale. Int J Health Geogr.

[27] Jacquez G, Greiling D, Kaufmann A (2005). Design and implementation of a space-time intelligence system for disease surveillance. J Geographic Syst.

[28] Rogerson P, Yamada I (2008). Statistical detection and surveillance of geographic clusters.

[29] Kwan M-P (2012). The uncertain geographic context problem. Ann Assoc Am Geograph.

[30] Delmelle E, Dony C, Casas I, Jia M, Tang W (2014). Vizualizing the impact of space-time uncertainties on dengue fever patterns. Int J Geographic Inform Sci.

[31] Burra T, Jerrett M, Burnett R, Anderson M (2002). Conceptual and practical issues in the detection of local disease clusters: a study of mortality in Hamilton, Ontario. Can Geograph.

[32] Zimmerman D, Li J, Fang X (2010). Spatial autocorrelation among automated geocoding errors and its effects on testing for disease clustering. Stat Med.

[33] Zimmerman DL, Li J (2010). The effects of local street network characteristics on the positional accuracy of automated geocoding for geographic health studies. Int J Health Geograph.

[34] Harada Y, Shimada T (2006). Examining the impact of the precision of address geocoding on estimated density of crime locations. Comput Geosci.

[35] Bailey T, Gatrell Q (1995). Interactive spatial data analysis.

[36] Lemke D, Mattauch V, Heidinger O, Pebesma E, Hense H-W (2015). Comparing adaptive and fixed bandwidth-based kernel density estimates in spatial cancer epidemiology. Int J Health Geographic.

[37] Silverman BW (1986). Density estimation for statistics and data analysis.

[38] Amaral S, Gavlak AA, Escada MIS, Monteiro AMV (2012). Using remote sensing and census tract data to improve representation of population spatial distribution: case studies in the Brazilian Amazon. Popul Environ.

[39] Lemke D, Mattauch V, Heidinger O, Pebesma E, Hense HW (2013). Detecting cancer clusters in a regional population with local cluster tests and Bayesian smoothing methods: a simulation study. Int J Health Geogr.

[40] Delmelle E, Delmelle EC, Casas I, Barto T (2011). H.E.L.P: a GIS-based health exploratory analysis tool for practitioners. Appl Spatial Anal Policy.

[41] Dinas Komunikasi dan Infomatika (2017). Bidang Data dan Statistik: Open Data Kota Bandung.

[42] World Health Organization (2011). Comprehensive guidelines for prevention and control of dengue and dengue haemorrhagic fever.

[43] Bithell J (1990). An application of density estimation to geographical epidemiology. Stat Med.

[44] Hazelton ML (2017). Testing for changes in spatial relative risk. Stat Med.

[45] Wu TJ, Chen CF, Chen HY (2007). A variable bandwidth selector in multivariate kernel density estimation. Stat Prob Lett.

[46] Jaya IGNM, Folmer H. Identifying spatiotemporal clusters by means of agglomerative hierarchical clustering and bayesian regression analysis with spatiotemporally varying coefficients: methodology and application to dengue disease in Bandung, Indonesia. Geographic Anal. 2020. 10.1111/gean.12264.

[47] Kalinic M, Krisp JM (2018). Kernel Density Estimation (KDE) vs. Hot-Spot Analysis – Detecting Criminal Hot Spots in the City of San Francisco.

[48] Astuti EP, Dhewantara PW, Prasetyowati H, Ipa M, Herawati C, Hendrayana K (2019). Paediatric dengue infection in Cirebon, Indonesia: a temporal and spatial analysis of notified dengue incidence to inform surveillance. Parasites Vectors.

[49] Corwin AL, Larasati RP, Bangs MJ, Wuryadi S, Arjoso S, Sukri N, Listyaningsih E, Hartati S, Namursa R, Anwar Z, Chandra S, Loho B, Ahmad H, Campbell JR, Porter KR (2001). Epidemic dengue transmission in southern Sumatra, Indonesia. Trans R Soc Trop Med Hyg.

[50] Dhewantara PW, Marina R, Puspita T, Ariati Y, Purwanto E, Hananto M, Hu W, Soares Magalhaes RJ (2019). Spatial and temporal variation of dengue incidence in the island of Bali, Indonesia: An ecological study. Travel Med Infect Dis.

[51] Tosepu R, Tantrakarnapa K, Nakhapakorn K, Worakhunpiset S (2018). Climate variability and dengue hemorrhagic fever in Southeast Sulawesi Province, Indonesia. Environ Sci Pollut Res.

[52] Polwiang S (2020). The time series seasonal patterns of dengue fever and associated weather variables in Bangkok (2003-2017). BMC Infect Dis.

[53] Choi Y, Tang CS, McIver L, Hashizume M, Chan V, Abeyasinghe RR, Iddings S, Huy R (2016). Effects of weather factors on dengue fever incidence and implications for interventions in Cambodia. BMC Public Health.

[54] Chien LC, Yu HL (2014). Impact of meteorological factors on the spatiotemporal patterns of dengue fever incidence. Environ Int.

[55] Anker M, Arima Y (2011). Male-female differences in the number of reported incident dengue fever cases in six Asian countries. Western Pac Surveill Response J.

[56] Antony J, Celine TM (2014). A descriptive study on dengue fever reported in a Medical College Hospital. Sahel Med J.

[57] Eong OE (2001). Changing pattern of dengue transmission in Singapore. Dengue Bullet.

[58] BPS & KPPPA (2017). Pembangunan Manusia Berbasis Gender 2017.

[59] Chang CJ, Chen CS, Tien CJ, Lu MR (2018). Epidemiological, clinical and climatic characteristics of dengue fever in Kaohsiung City, Taiwan with implication for prevention and control. PLoS One.

[60] Kosasih H, Alisjahbana B, Nurhayati MQ, Rudiman I, Widjaja S, Antonjaya U, et al. The epidemiology, virology and clinical findings of dengue virus infections in a cohort of indonesian adults in western java. PLoS Negl Trop Dis. 2016. 10.1371/journal.pntd.0004390. 10.1371/journal.pntd.0004390 PMC4752237 26872216

[61] Sasmono RT, Taurel AF, Prayitno A, Sitompul H, Yohan B, Hayati RF, Bouckenooghe A, Hadinegoro SR, Nealon J (2018). Dengue virus serotype distribution based on serological evidence in pediatric urban population in Indonesia. PLoS Negl Tropical Diseases..

[62] Rodriguez-Barraquer I, Cordeiro MT, Braga C, de Souza WV, Marques ET, Cummings DA (2011). From re-emergence to hyperendemicity: the natural history of the dengue epidemic in Brazil. PLoS Negl Trop Dis.

[63] Paramita B (2016). The land-use of Bandung, its density, overcrowded area and public facility toward a compact city. IOP Conference Series: Materials Science and Engineering.

[64] Aziz S, Ngui R, Lim YA, Sholehah I, Nur Farhana J, Azizan AS, Wan Yusoff WS (2012). Spatial pattern of 2009 dengue distribution in Kuala Lumpur using GIS application. Trop Biomed.

[65] Dickin SK, Schuster-wallace CJ, Elliott SJ (2013). Developing a vulnerability mapping methodology: applying the water-associated disease index to dengue in Malaysia. PLoS One.

[66] Gubler DJ, Greenwood B, De Cock K (1998). Population growth, urbanization, automobiles and airplanes: the dengue connection. New and Resurgent Infections: Prediction, Detection and Management of Tomorrow’s Epidemics.

[67] Gubler DJ (2011). Dengue, Urbanization and Globalization: The Unholy Trinity of the 21st Century. Trop Med Health.

[68] Messina JP, Brady OJ, Golding N, Kraemer MUG, Wint GRW, Ray SE, Pigott DM, Shaerer FM, Johnson K, Earl L, Marczak LB, Shirude S, Weaver ND, Gilbert M, Velayudhan R, Jones P, Jaeisch T, Scott TW, Reiner RC, Hay SI (2019). The current and future global distribution and population risk of dengue. Nat Microbiol.

[69] Sarfraz MS, Tripathi NK, TIpdecho T, Thongbu T, Kerdthong P, Souris M (2012). Analyzing the spatio-temporal relationship between dengue vector larval density and land-use using factor analysis and spatial ring mapping. BMC Public Health.

[70] Warlina L, Guntara R (2019). Agricultural land use change into tourism area in Lembang Sub-district, West Bandung Regency, West Java Province, Indonesia. IOP Conference Series: Materials Science and Engineering.

[71] Phanitchat T, Zhao B, Haque U, Pientong C, Ekalaksananan T, Aromseree A, Thaewnongiew K, Fustec B, Bangs MJ, Alexander N, Overgaard HJ (2019). Spatial and temporal patterns of dengue incidence in northeastern Thailand 2006-2016. BMC Infect Dis.

[72] Churakov M, Villabona-Arenas CJ, Kraemer MUG, Salje H, Cauchemez S (2019). Spatio-temporal dynamics of dengue in Brazil: Seasonal travelling waves and determinants of regional synchrony. PLOS Negl Trop Dis.

[73] Guzetta G, Marques-Toledo CA, Rosa R, Teixeira M, Merler S (2018). Quantifying the spatial spread of dengue in a non-endemic Brazilian metropolis via transmission chain reconstruction. Nat Commun.

[74] Eder M, Cortes F, Filha NTS, Franca GVA, Degroote S, Braga C, Ridde V, Martelli CMT (2018). Scoping review on vector-borne diseases in urban areas: transmission dynamics, vectorial capacity and co-infection. Infect Dis Poverty.

[75] Alex C, Djatnika S, Ridad A, Marcvan S, Robert C (2001). Hospital based clinical surveillance for dengue haemorrhagic fever in Bandung, Indonesia 1994–1995. Acta Trop.

[76] Tapia-Conyer R, Méndez-Galván J, Burciaga-Zúñiga P (2012). Community participation in the prevention and control of dengue: the patio limpio strategy in Mexico. Paediatr Int Child Health.

